# Lack of *Porcine circovirus 4* Genome Detection in Pig Samples from Italy and Spain

**DOI:** 10.3390/pathogens9060433

**Published:** 2020-05-31

**Authors:** Giovanni Franzo, Albert Ruiz, Laura Grassi, Marina Sibila, Michele Drigo, Joaquim Segalés

**Affiliations:** 1 Department of Animal Medicine, Production and Health (MAPS), University of Padua, 35020 Legnaro, Italy; laura.grassi.2@phd.unipd.it (L.G.); michele.drigo@unipd.it (M.D.); 2 IRTA, Centre de Recerca en Sanitat Animal (CReSA, IRTA-UAB), Campus de la Universitat Autònoma de Barcelona, 08193 Bellaterra, Spain; albert.ruiz@irta.cat (A.R.); marina.sibila@irta.cat (M.S.); 3 OIE Collaborating Centre for the Research and Control of Emerging and Re-Emerging Swine Diseases in Europe (IRTA-CReSA), 08193 Bellaterra, Spain; joaquim.segales@irta.cat; 4 Departament de Sanitat i Anatomia Animals, Universitat Autònoma de Barcelona (UAB), 08193 Bellaterra, Spain; 5 Centre de Recerca en Sanitat Animal (CReSA, IRTA-UAB), UAB, Campus de la Universitat Autònoma de Barcelona, 08193 Bellaterra, Spain

**Keywords:** PCV-4, Italy, Spain, molecular epidemiology, absence

## Abstract

The genus *Circovirus* includes several species and mostly causes asymptomatic infections. *Porcine circovirus 2* (PCV-2) and, with increasing evidence, *Porcine circovirus 3* (PCV-3), have been associated with different clinical conditions all over the world. In 2019, a new porcine circovirus (PCV-4) was identified from diseased animals in China. Because of the lessons learned from PCV-2 and PCV-3, it appears mandatory to investigate the actual distribution of this new virus and its potential association with clinical outcomes. To this purpose, an exploratory study to detect PCV-4 by molecular methods was performed in Italy and Spain by testing more than 300 samples of different types (serum and tissues), collected from both healthy and diseased pigs and wild boar as well. All samples, independently from the country, type, health status and host, tested PCV-4 negative. Therefore, no evidence of PCV-4 presence was found in Italy and Spain through this exploratory study. Considering the dense pig trade among European countries, its presence in the continent can similarly be considered unlikely. The reasons behind the restricted PCV-4 distribution compared to other porcine circoviruses will require further investigations. Careful surveillance might nevertheless be important since prompt recognition of PCV-4 would allow the implementation of effective countermeasures to prevent its spreading and potential economic losses.

## 1. Introduction

The genus *Circovirus* includes a group of single-stranded DNA viruses (ssDNA) with a circular genome of approximately 2 kb. The first circovirus identification report dates back to the early 1970s [1], when the virus subsequently named *Porcine circovirus 1* (PCV-1) was identified as a contaminant of PK-15 cell lines. The pathogenicity of some *Circovirus* members was at first demonstrated in birds during the 1980s and 1990s, when *Beak and feather disease virus* (BFDV), *Pigeon circovirus* (PiCV) and *Goose circovirus* (GoCV) were recognized as responsible for relevant diseases in the corresponding animal species [2]. However, the interest around this genus remained limited due to the marginal economic relevance of the involved hosts. Currently, 39 viral species have been classified within this genus, *Circovirus*, infecting avian and mammals and, to a much lesser extent, freshwater fishes and even ticks (https://talk.ictvonline.org/). None of these viral species had a significant impact on animal health, with the remarkable exception of *Porcine circovirus 2* (PCV-2) and, presumably, 3 (PCV-3).

PCV-2 was first identified in the middle of the 1990s and was thereafter reported all over the world, becoming the cause of one of the most relevant swine diseases, especially until the introduction of effective vaccines against this agent. A number of clinical conditions have been linked to PCV-2 infection, collectively named porcine circovirus diseases (PCVD), which can be broadly divided into systemic diseases and apparatus specific ones (reviewed in Segalés, 2012) [3].

PCV-3, although genetically distant from PCV-2, has followed a relatively comparable trajectory. It was recently identified by a metagenomic approach in 2015 in the USA from animals presenting clinical signs compatible with porcine dermatitis and nephropathy syndrome (PDNS) and reproductive failure [4]. Thereafter, it has been reported worldwide in animals showing several clinical outcomes, such as respiratory, reproductive, gastrointestinal and neurological disorders [5,6,7].

Despite less stringent evidence currently being available on its pathogenic role, a strong suspicion of association with reproductive disease and multisystemic inflammation is mounting [8]. Both PCV-2 and PCV-3 are frequently detected in asymptomatic animals [3,5,9,10], testifying the multifactorial nature of the disease and the need of co-factors (environmental, managerial, co-infections and host-related ones) for overt clinical signs development [11].

Recently, a fourth porcine circovirus (PCV-4) has been identified and reported only once in China from pigs displaying severe clinical disease like respiratory and enteric signs as well as PDNS [12]. Because of the lessons learned from PCV-2 and PCV-3, the knowledge of new porcine circoviruses, their distribution, epidemiology and putative disease association appear to be of pivotal importance. To this purpose, an exploratory study to detect PCV-4 by molecular methods was performed on samples obtained from pigs with different clinical conditions and collected in the densely pig populated areas of Northern Italy and Catalonia.

## 2. Results and Discussion

A total of 108 Spanish samples were included in the study. Particularly, 73 individual swine serum samples were obtained from 4-week- to 4-month-old pigs clinically diagnosed with respiratory signs (including interstitial/necrotizing pneumonia and pleuritis) (n = 30), and animals displaying enteric signs like atrophy-fusion of villi and catarrhal enteritis (n = 30) as well as healthy animals (n = 13). These samples were part of a set processed in a previously published article (Saporiti et al., 2020) [13]. Fifteen fetal tissue samples (eight from brains and seven from lungs) were also analyzed. These samples belonged to eight fetuses (three stillborn and five mummified) obtained from a farm within the Spanish standard productive and reproductive parameters. Finally, 20 porcine umbilical cord (PUC) pools, each containing serum from three piglets that came from healthy sows at farrowing, were tested (Table 1). The sows originated from a Spanish farm with standard productive and reproductive parameters.

The Italian study included 163 animals whose samples were obtained in the period 2013–2018 for diagnostic purposes from farms located in Northern Italy and previously reported in PCV-2 and PCV-3 studies [9,14,15,16]. Forty-five serum samples were collected from healthy animals, while the remaining samples corresponded to pigs with a variety of clinical signs. Twenty-one subjects showed respiratory signs, 34 evidence of systemic disease and 4 PDNS. The precise clinical outcome was not available for the remaining pigs (n = 59), although the disease status was reported (herein named “unknown disease”). Overall, 26 lymph nodes, 29 lungs, 60 sera and 48 other viscera were tested (Table 1). The sera of 100 wild boar, collected from Colli Euganei Regional Park [17] and Friuli Venezia Giulia alps [18], were also included in the study.

All samples, independently of the considered matrix, health status, host and country, tested PCV-4 negative. Several hypotheses could explain the observed scenario. At first, technical and experimental design limitations should be discussed. A low assay sensitivity or reaction inhibition could lead to false-negative results. However, the qPCR performances could be considered adequate, being able to detect up to 10–100 copies/μL of viral DNA, depending on the involved laboratory. Studies on other porcine circoviruses typically reported much higher viral loads especially, but not only, in diseased animals [19,20,21]. The occurrence of inhibition or poor extraction efficiency could also be excluded due to the inclusion of an internal control (IC) during sample pre-processing.

Recognizing the substantial lack of data on PCV-4 tropism, the inclusion of non-target tissues could have similarly affected our detection capability. Nevertheless, a broad range of tissues was tested in both countries, with a certain focus on those where PCV-4 was previously identified [12]. Similarly, a broad spectrum of clinical conditions, from healthy subjects to systemically diseased ones, were screened. Therefore, the features of the experimental study supported the robustness and consistency of the obtained results.

Consequently, an actual lack of PCV-4 circulation in Italy and Spain should be considered. Based on the available sample sizes and the assumed specificity and sensitivity, the probability of zero positive samples from a population with a minimum prevalence of 1% would be 0.0013 and <0.0001 in Spain and Italy, respectively. It is, therefore, possible to conclude that the populations are free from infection (at the considered threshold of 1% prevalence) with a 99.87% and ~100% confidence. Unfortunately, due to the absence of characterized PCV-4 samples, the actual test diagnostic sensitivity and specificity could not be experimentally estimated. However, the proposed values appear reasonable or even conservative based on previous PCV data [22]. While a circulation at extremely low prevalence cannot be excluded, it would conflict with the typical PCV epidemiology (PCVs are considered ubiquitous) and with the Chinese report [12].

If PCV-4 was present in other European countries, such assessment would require further investigations. However, also in this case, the dense pig trade among countries and data available for PCV-2 [23,24] and PCV-3 [7] make this hypothesis unlikely. This would be emphasized by the fact that tested animals corresponded to two of the most heavily swine populated areas in Europe.

Differently from other PCVs, and in absence of other reports at present, PCV-4 appears to have a limited geographic distribution, potentially restricted to China. Interestingly, while officially reported in 2019 [12], sequences of the *Rep* gene of PCV-4 were already reported by Biao He from pigs sampled in 2017 in China in the context of an unpublished work (“A reference catalog of the industrialized pig virome”, GenBank accession numbers MK377675.1, and MK948417.1-MK948424.1). Therefore, a prolonged PCV-4 presence (at least from 2017 to 2019) in China can apparently be confirmed.

As a side note, the abovementioned sequences share a high percentage of identity with those reported by Zhang et al., suggesting a certain viral stability over time and lessening another technical problem that could have affected the PCR assay sensitivity, i.e., the presence of mismatches between target and primers/probe. The presence of significantly heterogeneous viral strains in a different part of the world cannot be omitted. Nevertheless, this hypothesis would also conflict with the previous PCV experiences since these viruses have been found ubiquitously in the worldwide swine population [5,7,24,25]. Additionally, differently from other porcine pathogens like Porcine reproductive and respiratory syndrome virus [26], PCV-2 variability has not represented a major challenge for its diagnosis, especially when *Rep* targeting PCR assays were used.

If the limited PCV-4 distribution is due to its recent emergence or to other epidemiological factors remains to be established. Although present data do not point out the virus circulating in Europe, careful surveillance might be of interest. If PCV-4 is really able to cause disease and, by extension, economic problems in the swine industry, its prompt recognition would allow the implementation of effective countermeasures to prevent its spreading.

## 3. Material and Methods

### 3.1. Sampling Design

The only available PCV-4 study reported a 12% prevalence among randomly selected pigs in Hunan province, China. Therefore, with the aim of detecting at least one positive sample assuming a test specificity and sensitivity of 95%, prevalence of 10%, type I error of 5% and power of 80%, a sample size of 50 subjects was established as the lower acceptable limit. Nevertheless, a higher sample number was tested in order to increase the study sensitivity and to include a representative variety of tissue matrices and clinical conditions. To evaluate potential alternative hosts, the sera of 100 wild boar, collected from Colli Euganei Regional Park [17] and Friuli Venezia Giulia alps [18], were also included in the study. All included swine samples were collected in the context of routine diagnostic activities and/or were archive samples. No experimental treatments were implemented during the study, therefore approval of the ethics committee was not required

### 3.2. Diagnostic Assay Validation

The qPCR assay described by Zhang et al. [12] was used to detect PCV-4 genome in selected samples. Laboratory validation was performed in the Italian laboratory and, once optimized, transferred to the Spanish one. Since no virus isolate is currently available, the complete *Rep* gene, where primers and probes are located, was chemically synthesized (GenScript Biotech, Piscataway, NJ, USA) and cloned in a pUC57-Kan plasmid. The plasmid DNA was quantified (Qubit instrument, Thermo Fisher Scientific, Waltham, MA, USA) and the number of viral copies (i.e., plasmid) per μL was then calculated (DNA Copy Number and Dilution Calculator tool, https://goo.gl/ANXpex). A ten-fold dilution ranging from 1.14 × 10^9^ to 1.14 copies/μL was created. To emulate the physical–chemical features of the tested matrices, the dilution series was performed on lung homogenate. However, to prevent the risk of using a PCV-4 infected tissue, horse lung was used instead of a swine one. From each dilution level, DNA was extracted using the MagMax-96 Pathogen RNA/DNA kit (Applied Biosystem™, Foster, CA, USA) according to the manufacturer′s instructions. Just before extraction, 3 μL of QuantiNova Internal control (QN-IC) (QuantiNova^®^ Pathogen + IC Kit, Quiagen, Hilden, Germany) was added to the lung homogenate. Different primer and probe concentrations, as well as thermal protocols, were attempted to maximize assay sensitivity (Limit of Detection; LoD) and efficiency. The final settings, guaranteeing a LoD of 11.1 copies/μL and an efficiency of 99%, were the following:

Two microliters of DNA was added to a standard mix including 1× Pathogen Master Mix, 0.6 μM and 0.3 each of primer and probe and 1 μL of QuantiNova IC Probe Assay 10× (for IC detection). Molecular biology grade water was added up to a final volume of 10 μL. The cycling parameters were 95 °C for 7 min, followed by 45 cycles at 95 °C for 5 s and 60 °C for 30 s.

### 3.3. Sample Processing

All samples were processed using a common protocol in the two countries.

Two hundred microliters of serum were spiked with 3 μL of QN-IC and extracted using the MagMax-96 Pathogen RNA/DNA kit (Applied Biosystem™). One gram of tissue sample was mechanically homogenate in 9–10 mL of PBS or DMEM, and centrifuged at 2000 *g* for 2 min or 1000 *g* for 10 min at room temperature, and the DNA was extracted with the MagMax-96 Pathogen RNA/DNA kit from 200 μL of supernatant after spiking with 3 μL of QN-IC. All samples and DNA were stored at −20 °C until processing.

DNA was tested for PCV-4 genome presence using the abovementioned qPCR assay. The 7500 Fast Real-Time PCR System™ (Applied Biosystems) and LightCycler96 instrument (Roche Diagnostic) were used in Spain and Italy, respectively.

## Figures and Tables

**Table 1:** Summary of the features of the samples included in the Spanish and Italian study.

Country	Origin of Tested Samples	Type of Sample	N.
**SPAIN**	Healthy	Serum	13
	Enteric	Serum	30
	Respiratory	Serum	30
	PUC	Serum	20
	Stillborn/mummified	Tissue homogenates (lung and/or brain)	15
**ITALY**	Healthy	Serum	45
	PDNS	Tissue homogenates *	4
	Respiratory	Tissue homogenates * and serum	15
		Serum	6
	Systemic	Tissue homogenates *	25
		Serum	9
	Unknown disease	Tissue homogenates *	59

* Tissue homogenates contained lung, lymph nodes and/or other viscera.

## References

[1] Tischer I., Rasch R., Tochtermann G. (1974). Characterization of papovavirus-and picornavirus-like particles in permanent pig kidney cell lines. Zentralbl Bakteriol Orig A.

[2] Todd D. (2004). Avian circovirus diseases: Lessons for the study of PMWS. Veterinary Microbiology.

[3] Segalés J. (2012). Porcine circovirus type 2 (PCV2) infections: Clinical signs, pathology and laboratory diagnosis. Virus Res..

[4] Palinski R., Piñeyro P., Shang P., Yuan F., Guo R., Fang Y., Byers E., Hause B.M. (2017). A Novel Porcine Circovirus Distantly Related to Known Circoviruses Is Associated with Porcine Dermatitis and Nephropathy Syndrome and Reproductive Failure. J. Virol..

[5] Klaumann F., Correa-Fiz F., Franzo G., Sibila M., Núñez J.I., Segalés J. (2018). Current knowledge on Porcine circovirus 3 (PCV-3): A novel virus with a yet unknown impact on the swine industry. Front. Vet. Sci..

[6] Franzo G., He W., Correa-Fiz F., Li G., Legnardi M., Su S., Segalés J. (2019). A Shift in *Porcine Circovirus* 3 (PCV-3) History Paradigm: Phylodynamic Analyses Reveal an Ancient Origin and Prolonged Undetected Circulation in the Worldwide Swine Population. Adv. Sci..

[7] Franzo G., Delwart E., Fux R., Hause B., Su S., Zhou J.Y., Segalés J. (2020). Genotyping porcine circovirus 3 (PCV-3) Nowadays: Does it make sense?. Viruses.

[8] Arruda B., Piñeyro P., Derscheid R., Hause B., Byers E., Dion K., Long D., Sievers C., Tangen J., Williams T. (2019). PCV3-associated disease in the United States swine herd. Emerg. Microbes Infect..

[9] Franzo G., Legnardi M., Tucciarone C.M., Drigo M., Klaumann F., Sohrmann M., Segalés J. (2018). Porcine circovirus type 3: A threat to the pig industry?. Vet. Rec..

[10] Zheng S., Wu X., Zhang L., Xin C., Liu Y., Shi J., Peng Z., Xu S., Fu F., Yu J. (2017). The occurrence of porcine circovirus 3 without clinical infection signs in Shandong Province. Transbound. Emerg. Dis..

[11] Segalés J., Kekarainen T., Cortey M. (2013). The natural history of porcine circovirus type 2: From an inoffensive virus to a devastating swine disease?. Vet. Microbiol..

[12] Zhang H.H., Hu W.Q., Li J.Y., Liu T.N., Zhou J.Y., Opriessnig T., Xiao C.T. (2019). Novel circovirus species identified in farmed pigs designated as Porcine circovirus 4, Hunan province, China. Transbound. Emerg. Dis..

[13] Saporiti V., Cruz T.F., Correa-Fiz F., Núñez J.I., Sibila M., Segalés J. (2020). Similar frequency of Porcine circovirus 3 (PCV-3) detection in serum samples of pigs affected by digestive or respiratory disorders and age-matched clinically healthy pigs. Transbound. Emerg. Dis..

[14] Franzo G., Tucciarone C.M., Dotto G., Gigli A., Ceglie L., Drigo M. (2015). International trades, local spread and viral evolution: The case of porcine circovirus type 2 (PCV2) strains heterogeneity in Italy. Infect. Genet. Evol..

[15] Franzo G., Tinello S., Grassi L., Tucciarone C.M., Legnardi M., Cecchinato M., Dotto G., Mondin A., Martini M., Pasotto D. (2020). Free to circulate: An update on the epidemiological dynamics of porcine circovirus 2 (PCV-2) in Italy reveals the role of local spreading, wild populations, and Foreign countries. Pathogens.

[16] Franzo G., Legnardi M., Hjulsager C.K., Klaumann F., Larsen L.E., Segales J., Drigo M. (2018). Full-genome sequencing of porcine circovirus 3 field strains from Denmark, Italy and Spain demonstrates a high within-Europe genetic heterogeneity. Transbound. Emerg. Dis..

[17] Franzo G., Tucciarone C.M., Drigo M., Cecchinato M., Martini M., Mondin A., Menandro M.L. (2018). First report of wild boar susceptibility to Porcine circovirus type 3: High prevalence in the Colli Euganei Regional Park (Italy) in the absence of clinical signs. Transbound. Emerg. Dis..

[18] Franzo G., Grassi L., Tucciarone C.M., Drigo M., Martini M., Pasotto D., Mondin A., Menandro M.L. (2019). A wild circulation: High presence of Porcine circovirus 3 in different mammalian wild hosts and ticks. Transbound. Emerg. Dis..

[19] Franzo G., Legnardi M., Centelleghe C., Tucciarone C.M., Cecchinato M., Cortey M., Segalés J., Drigo M. (2018). Development and validation of direct PCR and quantitative PCR assays for the rapid, sensitive, and economical detection of porcine circovirus 3. J. Vet. Diagn. Investig..

[20] Grau-Roma L., Hjulsager C.K., Sibila M., Kristensen C.S., López-Soria S., Enøe C., Casal J., Bøtner A., Nofrarías M., Bille-Hansen V. (2009). Infection, excretion and seroconversion dynamics of porcine circovirus type 2 (PCV2) in pigs from post-weaning multisystemic wasting syndrome (PMWS) affected farms in Spain and Denmark. Vet. Microbiol..

[21] López-Lorenzo G., Díaz-Cao J.M., Prieto A., López-Novo C., López C.M., Díaz P., Rodríguez-Vega V., Díez-Baños P., Fernández G. (2019). Environmental distribution of Porcine Circovirus Type 2 (PCV2) in swine herds with natural infection. Sci. Rep..

[22] Franzo G., Segalés J., Klaumann F., Legnardi M., Mweu M.M., Mahmmod Y.S. (2019). Diagnostic accuracy of two DNA-based molecular assays for detection of porcine circovirus 3 in swine population using Bayesian latent class analysis. Lett. Appl. Microbiol..

[23] Franzo G., Cortey M., Segalés J., Hughes J., Drigo M. (2016). Phylodynamic analysis of porcine circovirus type 2 reveals global waves of emerging genotypes and the circulation of recombinant forms. Mol. Phylogenet. Evol..

[24] Franzo G., Segalés J. (2018). Porcine circovirus 2 (PCV-2) genotype update and proposal of a new genotyping methodology. PLoS ONE.

[25] Grau-Roma L., Fraile L., Segalés J. (2011). Recent advances in the epidemiology, diagnosis and control of diseases caused by porcine circovirus type 2. Vet. J..

[26] Drigo M., Franzo G., Gigli A., Martini M., Mondin A., Gracieux P., Ceglie L. (2014). The impact of porcine reproductive and respiratory syndrome virus genetic heterogeneity on molecular assay performances. J. Virol. Methods.

